# Demonstration of *Anaplasma marginale* transmission to cattle through the intra-transstadial and by unfed larvae of *Rhipicephalus microplus* routes, with subsequent calculation of infection rates

**DOI:** 10.1007/s11250-026-05124-4

**Published:** 2026-06-04

**Authors:** Luciana Maffini Heller, Dina María Beltrán Zapa, Igor Maciel Lopes de Morais, Vanessa Ferreira Salvador, Luccas Lourenzzo Lima Lins Leal, Luiz Fellipe Monteiro Couto, Lídia Mendes de Aquino, Lucianne Cardoso Neves, Bianca Barbara Fonseca da Silva, Lorena Lopes Ferreira, Felipe da Silva Krawczak, Caio Marcio de Oliveira Monteiro, Welber Daniel Zanetti Lopes

**Affiliations:** 1 Centro de Parasitologia Veterinária, Escola de Veterinária e Zootecnia, Universidade Federal de Goiás, Goiânia, Goiás Brazil; 2 Departamento de Medicina Veterinária, Escola de Veterinária e Zootecnia, Universidade Federal de Goiás, Goiânia, Goiás Brazil; 3 Departamento de Medicina Veterinária Preventiva, Escola de Veterinária, Universidade Federal de Minas Gerais, Belo Horizonte, Minas Gerais Brazil; 4 Departamento de Biociências e Tecnologia, Instituto de Patologia Tropical e Saúde Pública, Universidade Federal de Goiás, Goiânia, Goiás Brazil

**Keywords:** Anaplasmosis, Cattle tick, Tick fever

## Abstract

The objective of this study was to assess the infection rate of *Anaplasma marginale* in cattle by *Rhipicephalus microplus*. Two experiments were conducted for this purpose. Experiment 1 evaluated the infection rate by the intrastadial and transstadial routes. Calves naturally infected with *A. marginale* were infested with *R. microplus* and were called “positive with ticks” (PWT). These PWT animals were housed together with *A. marginale*-negative, tick-free calves, called “negative and free-tick” (NFT) for 31 consecutive days. On day 31, the PWT animals were removed from the pens and returned to the farm/herd of origin. The NFT animals remained in the pens until day 84, and the possible infection rate of these calves with *A. marginale* by cattle ticks via the transstadial or intrastadial route was investigated (using blood smears, PCR, and ELISA). During this period, we evaluated the transfer rate of *R. microplus* from PWT to NFT animals. As a result of Experiment 1, only adult male and female ticks were found on the NFT animals. The transfer rate of ticks from PWTs to NFT calves was 0.10% (31/28464), and 70% (7 of 10), and all the NFT animals housed along with PWT animals became infected by *A. marginale.* In Experiment 2, the infection rate of *A. marginale* in calves by *R. microplus* larvae was carried out in two steps. The first step involved the use of the first tick generation larvae, which were used to infest (at 40,000 larvae per animal) five NFT calves. If any of these five NFT animals became infected with this rickettsia, fully engorged females detached from these animals were collected to perform the second step of the Experiment 2, conducted with the second-generation larvae. The second-generation larvae came from the first generation. Another five NFT calves were infested with 40,000 larvae/animal from the second tick generation. As a result of Experiment 2, 100% of NFT calves were infected by *A. marginale* due to infestation with *R. microplus* larvae of the first and second generations. This tick species has epidemiological importance in the transmission of *A. marginale* to calves by the intra-transstadial or unfed larval route.

## Introduction

*Anaplasma marginale* is the causative agent of anaplamosis, an infectious disease that affects cattle in tropical and subtropical regions. Transmission occurs mainly biologically by ticks, or mechanically through contaminated instruments such as reused syringes and needles (Reinbold et al. 2010), via mechanical transmission by blood-feeding flies (Alonso et al. 1992), as well as transplacental transmission (Costa et al. 2016).

Studies carried out in tropical regions show that dairy calves between three and seven months of age have an average of five relapses of the disease before acquiring immunity (Heller et al. 2022). Furthermore, in dairy animals, each relapse of the disease results in approximately 213.5 L less milk in the first lactation (De Moraes et al. 2024). Recently, outbreaks of tick fever agents have been described worldwide and are frequently associated with *A. marginale* infection (Almazán et al. 2008; Gonçalves et al. 2011; Costa et al. 2011; Amorim et al. 2014; Machado et al. 2015; Aktas and Özübek 2017; Bahia et al. 2020; Curtis et al. 2021; Leal et al. 2024). This scenario highlights the importance of carrying out studies that help to understand biological aspects that can trigger such outbreaks. One of the doubts regarding this subject is how the infection by *A. marginale* occurs.

In the case of the tick *R. microplus*, there are two main transmission routes: intrastadial transmission (in which an adult male tick becomes infected from a carrier bovine and subsequently transmits the infection to another bovine) and transstadial transmission (involving infection in one stage and transmission in the next stage). These routes are facilitated by the movement of this tick individuals among cattle within the same herd (Guglielmone 1995; Mason and Norval 1981; Uilenberg 1970; Panizza et al. 2022a), and their occurrence has been scientifically demonstrated (Uilenberg 1970; Aguirre et al. 1994). However, the exact proportions of these two pathways in the field remain uncertain.

Additionally, the transovarian transmission of *A. marginal*e by *R. microplus* has been confirmed by some researchers (Rosenbusch and Gonzalez 1923; Lopez and Vizcaino, 1992; Estrada et al. 2020; De la Fournière et al. 2023), while others have not confirmed this (Thompson and Roa 1978; Potgieter 1979; Ribeiro et al. 1996; Ruiz et al. 2005; Esteves et al. 2015; Panizza et al. 2022b). As previously mentioned, there is still some doubt as to the extent to which this transmission route occurs and when it happens.

The aspects described above highlight that even though science has evolved, more studies are still needed to clarify some aspects. This study aims to assess the infection rate of *R. microplus* (through the intrastadial or transstadial route, or via unfed larvae) in transmitting *A. marginale* to cattle, under controlled experimental conditions.

## Materials and methods

Two experiments were conducted. In both, the analysis of *A. marginale* was performed only in calves, not in ticks. Furthermore, needles and syringes were not shared between animals during the entire experimental period. In Experiment 1, the objective was to assess the transmission of *A. marginale* by *R. microplus*, specifically examining intrastadial and transstadial transmission. In the second experiment, the transmission of *A. marginale* by unfed larvae of *R. microplus* to cattle was evaluated. The infection rate in the two experiments was evaluated considering how many of the 10 *A. marginale*-negative calves per experiment became infected by *A. marginale* through the transfer of ticks between infected or uninfected calves (Experiment 1), or when exposed to unfed larvae (Experiment 2).

In both experiments, each animal was infested with approximately 40,000 *R. microplus* larvae, a number determined based on the findings of Andreotti et al. (2018). In this study performed under field conditions by these authors, larval challenge was estimated by combining tick counts in grazing Nelore and Brangus cattle with recovery rates obtained from controlled infestations. Animals were maintained in adjacent paddocks at a stocking rate of approximately 0.6 animal units per hectare (AU/ha). Using this model, Andreotti et al. (2018) estimated larval exposure of approximately 27,108 larvae/day/animal for Nelore cattle and at least 76,119 larvae/day/animal for Brangus cattle. Based on these findings, approximately 40,000 larvae per animal were used in the present study, since these values indicate that, under natural grazing conditions, cattle may be exposed to this number of *R. microplus* larvae within about 1.5 days for Nelore and less than one day for Brangus animals.

Furthermore, the experiment 1 and 2 were conducted at the animal experimentation shed of the School of Veterinary and Animal Science of Federal University of Goiás (EVZ-UFG), located in Goiânia, Goiás, Brazil. The stalls in this shed were constructed from bricks, with cement floors with area of 9 m^2^. They were also equipped with nylon screens (mesh: 3 mm) to protect the calves from attacks by hematophagous flies.

Throughout these experiments, we took precautions to assess the presence of any insects that might affect the study’s results. Around the animal experimentation facility, we placed four traps containing attractants designed to capture flies (Target^®^). The insects captured during the studies were subsequently quantified and identified using established taxonomic keys such as Curran (1965), Borror and White (1970), Mariconi et al. (1999), Carvalho and Ribeiro (2000), Guimarães et al. (2001), and Carvalho et al. (2002). The *R. microplus* and the *A. marginale* strains used in this study were obtained under field conditions from the EVZ-UFG dairy farm. The strains of *R. microplus* and* A. marginale* from EVZ-UFG used in the present study had been previously characterized in earlier investigations. The tick population was designated as the GYN strain by Nicaretta et al. (2023). The *A. marginale* strain was subjected to phylogenetic analysis and molecular identification by Leal et al. (2024), which identified haplotypes 1 (OR760278) and 2 (OR760277).

If any animal required salvage treatment against *A. marginale* in any of the experiments, this was performed when calves showed PCV values ≤ 20% and *A. marginale* bacteremia ≥ 3%. In this case, the calf was treated with enrofloxacin at 7.5 mg/kg, administered intramuscularly (Knetomax^®^ - Elanco Animal Health -Facury Filho et al. 2012). All calves that had these values of PCV, bacteremia for *A. marginale*, and more than 100 females of *R. microplus* detached or counted, received salvage treatment with an acaricide also (Potenty^®^, MSD Animal Health).

### Experiment 1: Intra and transstadial infection rate of *A. marginale* by *R. microplus*

In this experiment, two groups of animals were formed: calves naturally infected with a field isolate of *A. marginale* and artificially infested using a field population of *R. microplus*, designated “positive with ticks” (PWT), and *A. marginale*-negative calves free of ticks, called “negative free ticks” (NFT).

**Fig. 1:**
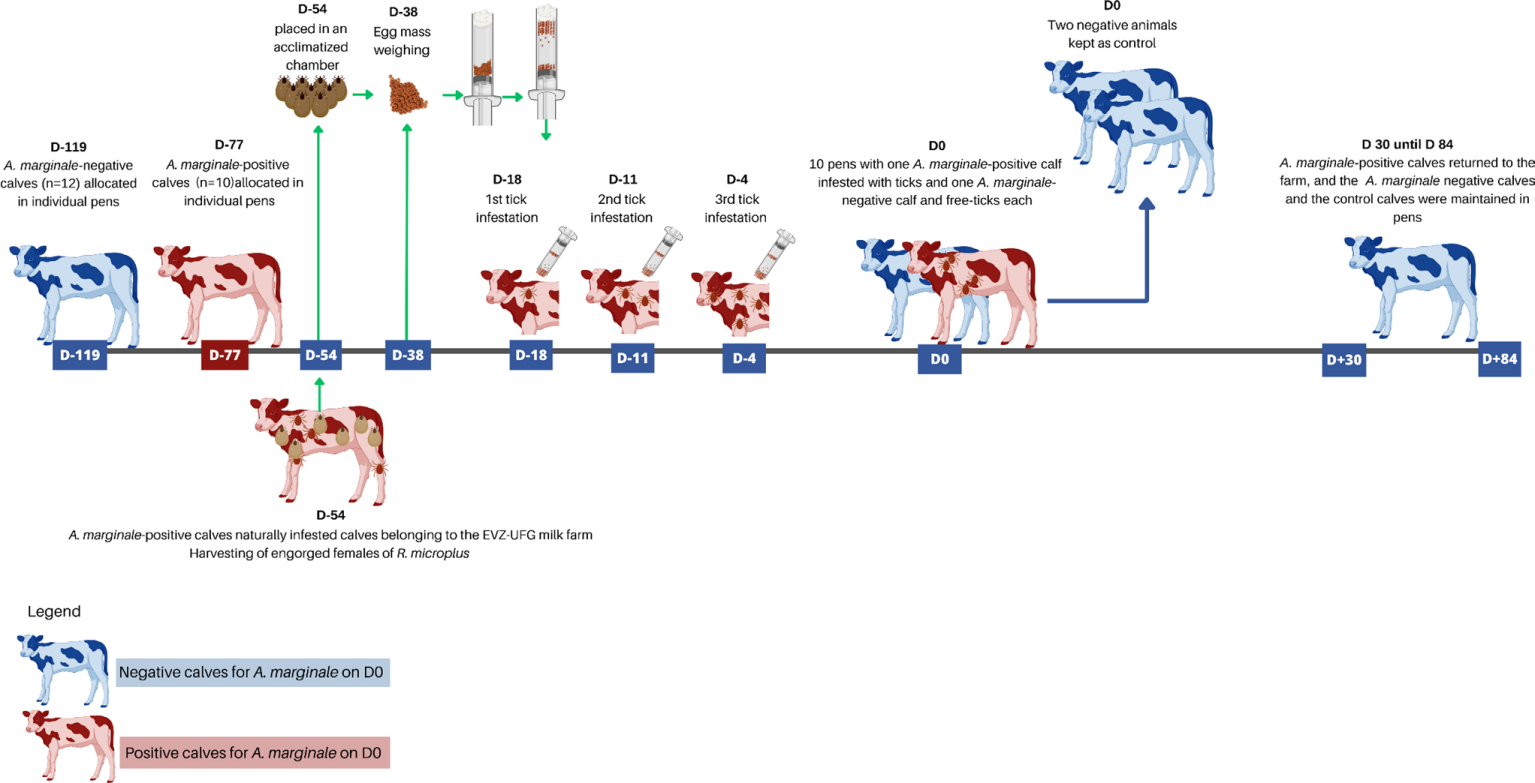
Experimental design adopted in Experiment 1 to evaluate the infection rate of *Anaplasma marginale* caused by transmission to cattle through the intra-transstadial route by *Rhipicephalus microplus*

Because the study was conducted in a region endemic for tick fever, we adopted strategies to ensure that the animals in the NFT group remained negative for *A. marginale* and *R. microplus*. Thus, the experiment began on day − 119 and continued until day 84. Day 0 of the study was defined as the day on which PWT calves were housed together with NFT calves (1:1). The NFT group arrived at the experimental facility on day − 119 since they were only one day old and needed to remain free from tick exposure until day 0, when they were housed together with the PWT group. The PWT animals naturally infected with a field isolate of *A. marginale* arrived at the facility on day − 77. Between day − 18 and day − 4, the PWT animals were infested with *R. microplus* larvae. In turn, from day 0 to day 30, NFT and PWT animals remained together. On day 30, the PWT animals were returned to the farm of origin. The NFT animals were kept in the pens and were monitored daily for the presence of ticks and *A. marginale* from day 0 to day 84. During the entire study, the stalls underwent cleaning twice a day, using a high-pressure sprayer. The experimental design is described in more detail below and is illustrated in Fig. 1.

### Experiment 1 - Negative calves for *A. marginale* (activities from day − 119 to day-1)

To select the NFT animals that would participate in Experiment 1, 16 newborn calves, with genetic proportion of ^15^/_16_ Holstein x ^1^/_16_ Gyr (Girolando), were acquired from a farm located in the municipality of Silvania, Goiás, Brazil. This farm is in an endemic area for *A. marginale*, but the cows and calves were placed in a free stall barn, without contact with *R. microplus*. Frozen colostrum was provided onsite at birth, and within approximately eight hours of life, all calves were transported to the experimentation shed at EVZ-UFG, where they were housed in individual pens.

When the 16 calves arrived, blood samples were collected from the coccygeal vein in tubes containing anticoagulant (K2EDTA, BD^®^). The samples were submitted to conventional polymerase chain reaction (PCR) for diagnosis of *A. marginale* (Torioni de Echaide et al. 1998, 2001), *Babesia bovis* and *Babesia bigemina* (De la Fournière et al. 2021; Parodi et al. 2021). Negative samples were further tested using PCR protocols targeting the cytochrome b (*cytB*) gene of mammals (Kocher et al. 1989) to validate the DNA extraction protocol. When any samples tested negative for these TF, additional PCR protocols were applied to verify the integrity of the DNA extraction procedure. Any sample failing to yield a product in this verification PCR was excluded from the study. The resulting PCR products were then stained with SYBR Safe (Invitrogen, Carlsbad, CA, USA), according to the manufacturer’s guidelines, and visualized via electrophoresis in a 1.5% agarose gel using an ultraviolet transilluminator.

One week after the calves arrived, 12 NFT animals were selected. Of these, 10 calves were denominated NFT and two as negative control. Regular PCR analyses were conducted at seven-day intervals from day − 112 and day − 1. Additionally, parasitological diagnoses for *A. marginale* were performed twice a week. This involved examining blood smears collected from the tip of the calves’ tails, stained with Giemsa, and observed under a microscope at 1000x magnification. The percentage of bacteremia for *A. marginale* was determined using the method outlined by IICA (1984) and Coetzee et al. (2006). The PCV measurements were taken twice a week, following the method described by Gomes et al. (2007). The packed cell volume (PCV) values falling outside the normal range, specifically ≤ 24% or ≥ 46%, were considered indicative of abnormal values according to the criteria provided by Feldman et al. (2000). To assess the presence of antibodies of the anti-*A. marginale* IgG class, serum samples were collected on days day − 7 and day − 1. The indirect ELISA technique (iELISA) was employed for this purpose, following the protocol described by Andrade et al. (2004).

The animals were bottle-fed with milk replacer (Nattimilk^®^-Auster) until weaning at 90 days old. After weaning, they received feed equivalent to about 1% of their body weight daily, along with corn silage and water *ad libitum*.

### Experiment 1 - Positive calves for *A. marginale(activities* from day − 77 to day − 1)

To compose the group of PWT animals, on day − 77, we selected 10 calves (^7^/_8_ Holstein x ^1^/_8_ Gyr), approximately 95 days old, naturally infected with *A. marginale* and naturally infested with *R. microplus*, originated from the EVZ-UFG dairy farm. This farm’s region is considered endemic for TF agents, with 100% of calves aged approximately 70 to 90 days being naturally infected with *A. marginale* (Heller et al. 2022). Confirmation of *A. marginale* infection in these 10 calves was carried out through blood smears (bacteremia ≥ 2%), as mentioned above, before being housed in the barn.

On day − 77, the 10 calves to compose the PWT group were housed in individual pens. They received preventive treatment against ticks (Potenty^®^, MSD Animal Health) and *Babesia* spp. (three applications at an interval of 24 h of diminazene 3.5 mg/kg, Ganazeg^®^ - Elanco Animal Health – Papich 2021). Between day − 77 and day − 1, PCV measurements and blood smears were conducted twice a week, as described earlier.

### Experiment 1 - Tick infestation in positive calves (activities from day − 54 to day 0)

The population of *R. microplus* came from the EVZ-UFG dairy farm, the same location where the 10 PWT calves were obtained.

On day − 54, fully engorged *R. microplus* females were collected directly from these naturally infested calves. It is important to highlight that the tick population used in this study was not derived from a colony maintained in a laboratory, nor from animals free of *A. marginale* and *Babesia* spp. Ticks exhibiting anomalies in size or shape were promptly discarded. The females were dorsally fixed individually in sterile plastic Petri dishes (9 centimeters in diameter) and placed in an acclimatized chamber (Biological Oxygen Demand – BOD) with temperature of 27 ± 1 °C and ≈ 85% relative humidity for oviposition. Fifteen days later (D-38), pools of 250 mg of eggs, corresponding to approximately 5,000 eggs (Labruna et al. 1997) were formed and transferred to adapted 3-mL syringes, then returned to the BOD chamber for larvae to hatch under the same controlled conditions.

On days − 18, -11, and − 4, when the larvae were approximately 7, 14, and 21 days old, each of the 10 calves from the PWT group was infested with approximately 15,000, 15,000, and 10,000 larvae of *R. microplus*, respectively (totaling 40,000 larvae per animal). The calves were restrained with a halter, and the larvae were applied to their dorsum using a syringe. The calves were kept restrained for 60 min to allow larval attachment. The tick infestations concluded on day − 4 to ensure that on day 0 of the study, practically all the larvae were already attached, following the method described by Roberts ([Bibr CR51][Bibr CR52]).

### Experiment 1 - Transmission study (activities from day 0 to day 84)

On day 0 of the study, a total of 11 pens (pens 1 to 11) were used. Each pen accommodated one PWT and one NFT animal, except for pen 11, which housed two negative control calves (negative for *A. marginale* and without infestation by *R. microplus*). Thus, one NFT animal was in direct contact with one PWT animal. The mean stocking rate in each pen was approximately 49.8 AU/hectare. The PWT and NFT calves shared the same pen for 31 days (day 0 to day 30). Throughout the study, all pens were cleaned twice daily to prevent detached ticks from oviposition and larvae from infesting the environment.

For PWT animals, daily tick counts (*R. microplus* females ≥ 4.5 mm in length) were conducted between day 0 and day 30. The counting method was adapted from Wharton and Utech (1970). The NFT cattle underwent daily counts for all *R. microplus* stages (male and female, larvae and nymphs) from day 0 to day 84. Until day 30, tick counts were first conducted on NFT calves and then on PWT calves to avoid tick transfer between animals during the counts. No ticks were physically removed from the calves during the study. Instead, only the number of ticks present was recorded at the time of counting. The pre-patent period was determined from the first observation of a tick on NFT calves and the presence of *A. marginale* in the blood smear, or DNA by PCR.

From both PWT and NFT calves, blood samples were collected daily between day 1 and day 30 to measure PCV (Gomes et al. 2007), *A.* *marginale* bacteremia (IICA, 1984; Coetzee et al. 2006). Additionally, NFT calves had weekly blood samples taken until day 30, for PCR analysis of *A. marginale msp5* (Torioni de Echaide et al. 1998, 2001).

On day 30, PWT calves were removed from the pens, received salvage treatment against ticks and *A. marginale*, and returned to the EVZ-UFG dairy farm herd. The NFT calves remained in the pens until day 84. During this period, blood samples for PCV and *A. marginale* bacteremia were collected twice a week, from day 35 to day 84. Blood samples for PCR analysis of *A. marginale msp5* (Torioni de Echaide et al. 1998, 2001) were performed weekly from day 35 to day 84. Additionally, serum samples were collected at the end of the study from each calf (day 45 for two NFT calves and day 84 for the others), to detect antibodies via ELISA (Andrade et al. 2004).

### Experiment 2: Transmission of *A. marginale* for calves by unfed *R. microplus* larvae

Activities performed in Experiment 2 went from day − 119 to day 84, and were performed in two steps: one with the first tick generation and the other with the second generation. Day 0 of the study was considered as the day when the NFT animals were infested with 40,000 tick larvae. The first groups of NFT animals used in step 1, arrived at the experimental facility on day − 119. The PWT animals, that came from the EVZ-UFG dairy farm, were naturally infected with a field isolate of *A. marginale* and naturally infested with a field population of *R. microplus*, arrived at the facility on day − 49. On day − 42, engorged females of this tick naturally detached of the PWT animals were used to produce the first tick generation. On this same day (day − 42), the PWT animals were returned to the farm of origin. First-generation tick larvae were obtained and used to infest five NFT animals on day 0 of the study.

**Fig. 2:**
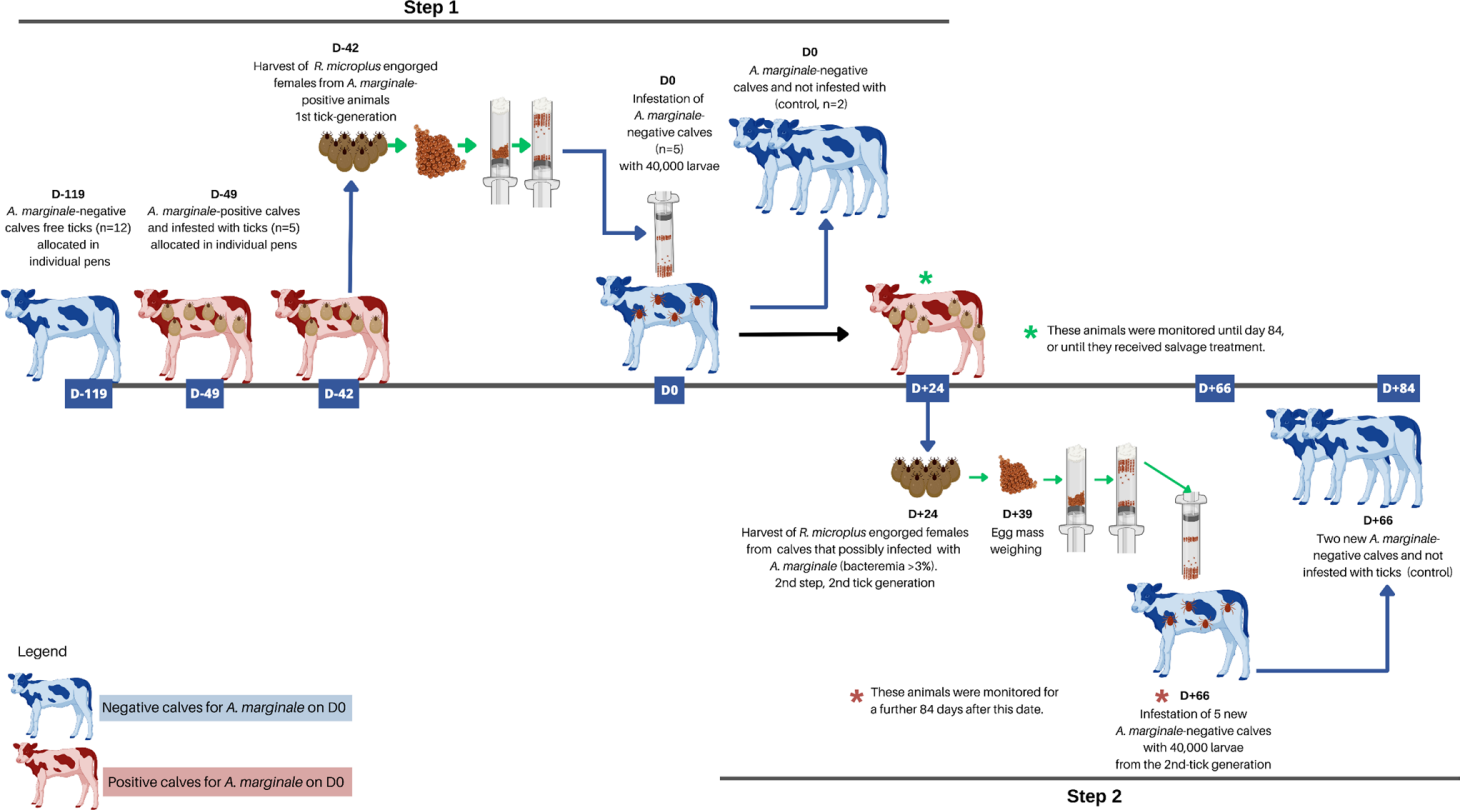
Experimental design adopted in Experiment 2 to evaluate the infection rate of *Anaplasma marginale* via transmission to cattle by larvae of *Rhipicephalus microplus*

From the NFT animals that were infected with *A. marginale*, engorged females of *R. microplus* were collected between day 20 and 25 to obtain the second-generation tick larvae. It is important to mention that, the second-generation larvae of ticks were obtained from the offspring of the first generation. These larvae belonging to the second generation were used to infest a second group of NFT animals between days 62 and 67. Both groups of NFT animals were kept in the pens, and were evaluated daily for up to 84 days post-infestation, except when an animal required salvage treatment against *A. marginale* or *R. microplus*. The experimental design is described in more detail below and illustrated in Fig. 2.

**Fig. 3:**
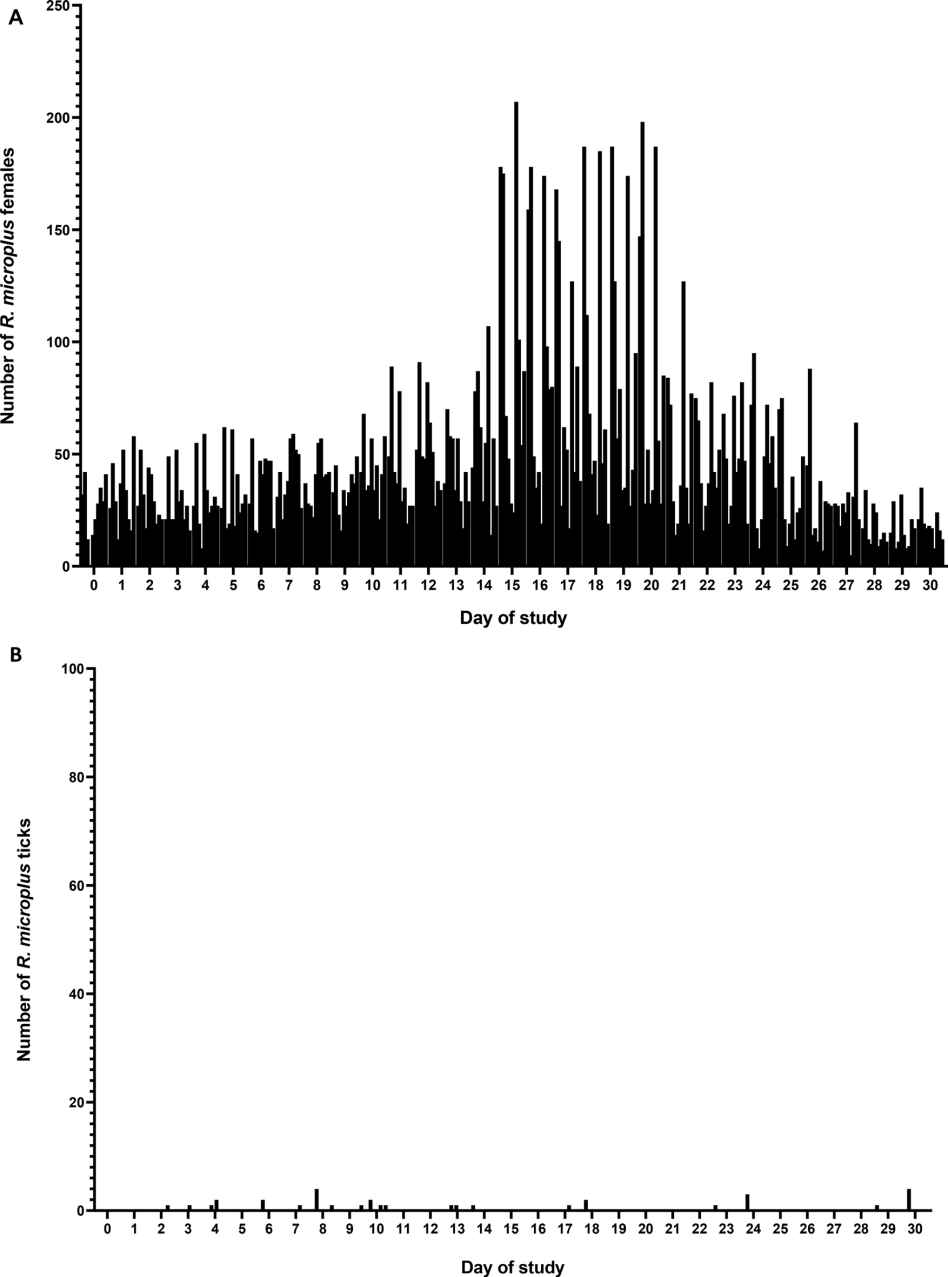
Tick counts in Experiment 1. **A**: Counts of *Rhipicephalus microplus* females (≥ 4.5 mm in length), from the 10 calves naturally infected by *Anaplasma marginale* and artificially infested with ticks (PWT) during D0 to D + 30; **B**: Tick counts (male and female) of *R. microplus* present in the 10 calves negative for *A. marginale* and free-ticks (NFT) that coexisted with the 10 PWT calves during D0 to D + 30

### Experiment 2: Step 1 - Negative calves for *A. marginale* (activities from day − 119 to day-1)

We obtained seven NFT calves for this experiment, and the experimental procedures were carried out until day − 1, following the same procedure described for Experiment 1.

### Experiment 2: Step 1 - Positive calves for *A. marginale* (activities from day − 49 to day-1)

Five PWT animals naturally infected with *A. marginale* and naturally infested with *R. microplus* were transported to pens on day − 49. 

On day − 42, engorged females of *R. microplus* naturally detached from these calves, with *A. marginale* bacteremia ≥ 3%, were brought to the laboratory. After that, the PWT animals were returned to the EVZ-UFG dairy farm (place of origin). One hundred and twenty engorged female ticks previously selected for showing good mobility and repletion were fixed in plastic Petri dishes and moved to an incubator (Biological Oxygen Demand – B.O.D) at temperature of 27 °C and approximately 85% relative humidity for oviposition. Fifteen days after oviposition in the incubator, the egg masses were homogenized and weighed in pools of 250 mg. On day − 27, each pool containing 250 mg of egg mass (approximately 5,000 eggs) was transferred to adapted 3 mL syringes and returned to the incubator for larval hatching under the same controlled conditions. After hatching, on day 0 of the study, the larvae were about 15 days old.

### Experiment 2: Step 1 – infesting NFT calves with the first tick generation, and collection of ticks from NFT animals that became infected with *A. marginale* (activities from day 0 to day 84)

On day 0 of the study, five NFT calves were infested individually with 40,000 unfed *R. microplus* larvae. Two calves were not infested and were kept as negative controls. The larval infestation was carried out by restraining the calves with halters and applying the larvae over their dorsum using a syringe. The calves were kept restrained for 60 min to allow the larvae to attach. Each of these seven calves remained isolated in individual pens until the study’s end on day 84.

Throughout the study, all pens were washed daily in the morning, typically between 08:00 and 09:00 a.m., using a high-pressure sprayer. Between day 20 and day 25, engorged females that detached from the NFT calves were collected from each animal, counted and stored.

Blood samples were taken daily from day 1 to day 84 to measure PCV and *A. marginale* bacteremia. In the same period, weekly blood samples were also taken to perform PCR for *A. marginale msp5* (Torioni de Echaide et al. 1998, 2001), and serum samples were collected weekly from each calf starting on day 14 for antibody measurement by ELISA (Andrade et al. 2004). Only on the last date that the animal remained in the study was the antibody test performed by ELISA.

### Experiment 2: Step 2 – the second generation of ticks

The engorged female ticks detached and collected on day 24 from calf #36 (mean *A. marginale* bacteremia of 3.8%) and calf #37 (mean *A. marginale* bacteremia of 7.2%), that became infected with *A. marginale* in step 1 produced the second generation of tick larvae used to perform step 2. For this purpose, concomitant with the execution of step 1, another seven NFT animals were obtained and kept onsite, awaiting the results of step 1. All the parasitological diagnostic procedures on the calves prior to infestation (PCR, ELISA, PCV, blood smear) were performed as described in step 1. The NFT calves from step 2 were infested on day 66 with 40,000 larvae belonging to the second generation. These animals (five infested and two kept as negative controls), were examined for *A. marginale* until 84 days after infestation with tick larvae belonging to the second generation (Fig. 2).

## Results

### Experiment 1

Between day 0 and day 30, 14,232 females of *R. microplus*, ≥ 4.5 mm in length, were counted in the 10 PWT calves. Among the 10 NFT calves, between day 2 and day 30, 31 adult ticks were observed, 29 males and two females. Notably, one of the 10 NFT calves did not have any ticks. In the nine NFT calves with observed ticks during this period, the maximum number of ticks observed per day ranged from 1 to 3 (see Table 1 and Fig. 3AB). Considering there is typically at least one male tick for each female, the transfer rate of *R. microplus* in NFT calves during the study was calculated to be 0.10% (31 out of 28,464 ticks).

**Table 1:** Summary of the results obtained in Experiment 1, to evaluate the intra and transstadial transmission rates of *Anaplasma marginale* by *Rhipicephalus microplus* in calves

Pen	Animal	Positive animal for *A. marginale* on Day 0?	Tick counts during the study	Days of the study that tick was found	Number of ticks found per day	NFT animal infected with *A. marginale*?	Pre-patent period* by blood smear	Mean of *A. marginale* bacteremia (range)	NFT animal that infected with *A. marginale* received salvage treatment? (Day - PCV - bacteremia)
1	1	No	3	14; 23; 29	1; 1; 1	No	-	0.0	No
	2	Yes	2033	0 up to 30	mean 67.8	-	-	0.82 (0.05–2.6)	-
2	3	No	0	not found	not found	No	-	0.0	No
	105	Yes	2377	0 up to 30	mean 79.2	-	-	2.94 (0.4–4.2)	-
3	4	No	16	01; 08; 10; 13; 18; 24	2; 3 and 1 engorged female; 3; 2; 2; 3	Yes	14	0.18 (0.0–0.6)	No
	63	Yes	977	0 up to 30	mean: 32.6	-	-	0.36 (0.0–1.8)	-
4	7	No	1	4	1	Yes	24	0.59 (0.0 3.6)	Yes (45 − 14% − 3.4%)
	12	Yes	876	0 up to 30	mean: 29.2	-	-	0.66 (0.0–2.6)	-
5	8	No	1	13	1	Yes	32	0.22 (0.0–0.6)	No
	91	Yes	1213	0 up to 30	mean: 40.4	-	-	0.38 (0.0–1.2)	-
6	9	No	3	03; 04	1; 1 and 1 engorged female	Yes	19	1.01 (0.1–5.0)	Yes (45 − 17% − 5.2%)
	119	Yes	1096	0 up to 30	mean: 36.5	-	-	1.39 (0.0–3.2)	-
7	10	No	3	07; 10; 17	1; 1; 1	Yes	22	0.02 (0.0–0.2)	No
	85	Yes	2086	0 up to 30	mean: 69.5	-	-	1.02 (0.2–3.2)	-
8	11	No	1	2	1	Yes	15	0.25 (0.0–0.6)	No
	98	Yes	1129	0 up to 30	mean: 37.6	-	-	0.73 (0.0–2.0)	-
9	13	No	2	08; 10	01; 01	Yes	20	0.04 (0.0–0.4)	No
	112	Yes	1217	0 up to 30	mean: 40.6	-	-	0.69 (0.0–2.1)	-
10	16	No	1	9	1	No	-	0.0	No
	424	Yes	1228	0 up to 30	mean: 40.9	-	-	1.23 (0.2–3.8)	-
11	CN1	No	0	not found	not found	No	-	0.0	-
	CN2	No	0			No	-	0.0	-

NFT = Negative calves for *A. marginale* and tick-free on day 0 of the study

PCV = Packed cell volume

* The pre-patent period was considered from the first time that a tick was found on the NFT and *A. marginale* in blood smears or DNA by PCR

Out of the 10 NFT calves, seven that had ticks became infected with *A. marginale* after being housed together with the PWT calves, resulting in an infection rate of 70% (7 out of 10). Blood smears indicated the presence of rickettsia in NFT calves after 14, 15, 19, 20, 22, 24, and 32 days (as shown in Table 1). The PCR revealed that six out of the seven NFT calves that were infected tested positive for *A. marginale* DNA on day 14. On day 21, all seven NFT calves showed positive PCR results for *A. marginale*. Furthermore, all seven NFT cattle also developed antibodies against *A. marginale* and had positive antibody titers at the end of the study (Table 2). Two of the seven NFT calves infected by *A. marginale* (animals 7 and 9) required salvage treatment with enrofloxacin on day 45, since their PCV values were ≤ 18% and bacteremia was ≥ 3% on that day (Table 1).

**Table 2:** Molecular detection of *Anaplasma marginale (msp5)* and antibody titers (ELISA) in blood or serum samples, respectively, collected from calves in Experiment 1

Group	Number of positive calves for *Anaplasma marginale* by PCR						
	Day − 119 to Day 7	Day 14	Day 21	Day 28	Day 35	Day 42	Day 49 up 84
NFT	0/10	6/10	7/10	7/10	7/10	7/10	5/7
PWT	10/10	Not performed		10/10	Not performed		
Negative controls	0/2	0/2	0/2	0/2	0/2	0/2	0/2
NFT	Antibody titers against *A. marginale* by ELISA, at the end of the study for each calf, of NFT calves and controls up to D0						
	Day − 7 and − 1					Day 45	Day 84
1	Negative						Negative
3	Negative						Negative
4	Negative						0.070 *
7	Negative					0.146 *	
8	Negative						0.787 *
9	Negative					0.987 *	
10	Negative						0.219 *
11	Negative						0.217 *
13	Negative						0.249 *
16	Negative						Negative
NC 1	Negative						Negative
NC 2	Negative						Negative

* Calves received salvage treatment against *A. marginale*

NFT = Negative calf for *A. marginale* and tick-free until day 0 of the study

PWT = Positive calf for *A. marginale* and infested with *R. microplus* on day 0 of the study

NC = Negative control

The two control cattle remained uninfected by *A. marginale* throughout the experimental period, as confirmed by blood smears, PCR and ELISA (Table 2). Additionally, they were not infested by ticks (Table 1). Flies belonging to the Calliphoridae family, as well as *Musca domestica* and phytophagous hemipterans, were found in traps to capture flies around the barn.

### Experiment 2

#### First tick generation

**Fig. 4:**
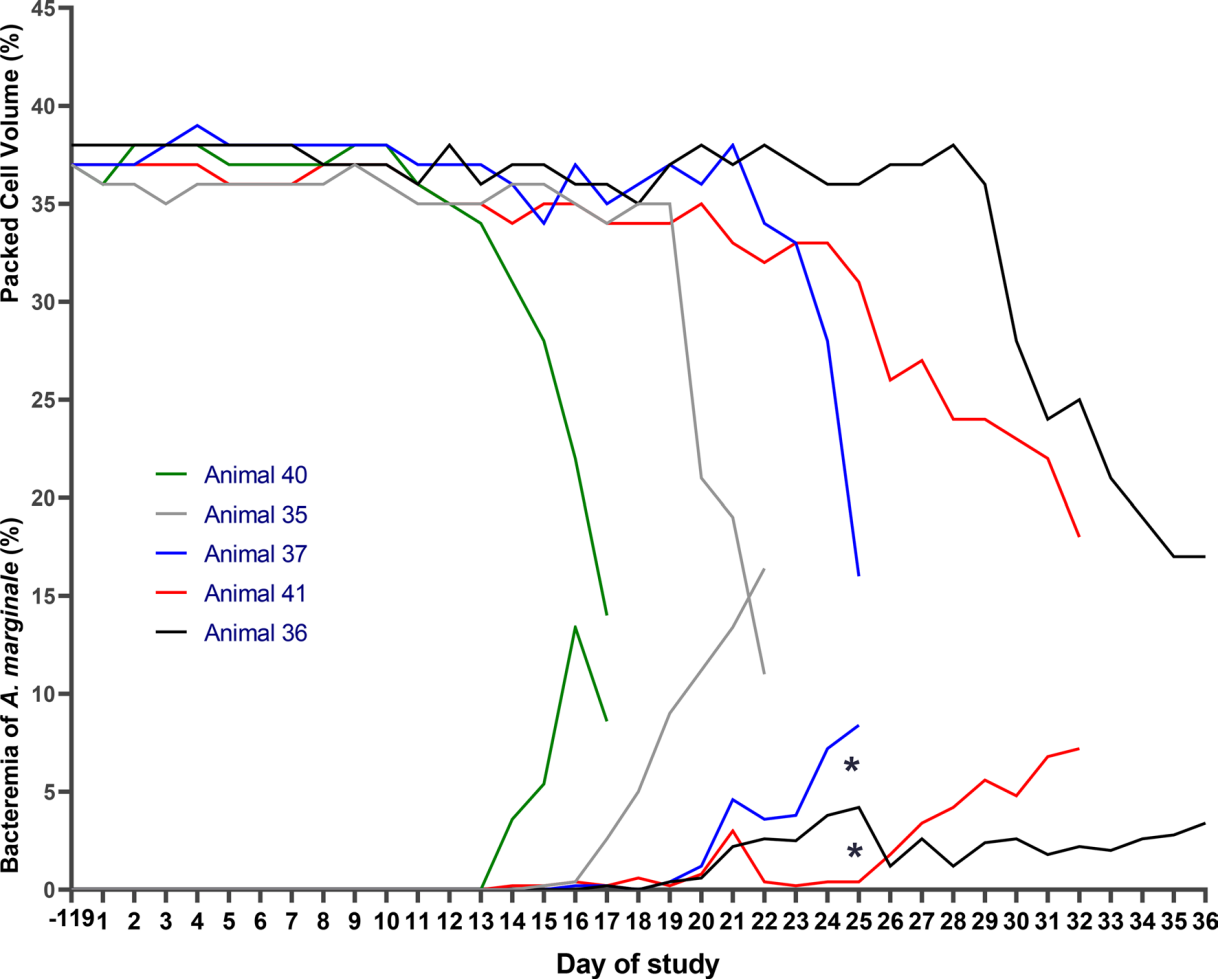
Experiment 2 - step 1: bacteremia of *Anaplasma marginale* and packed cell volume in calves negative for *Anaplasma marginale*, infested on day 0 of the study with 40,000 larvae of the first generation of *Rhipicephalus microplus* *= D + 24, the day when 224 engorged females of *R. microplus* from the calves were counted. Total of 36 (mean *A. marginale* bacteremia of 3.8%) and 37 (mean *A. marginale* bacteremia of 7.2%) ticks were collected for use in step 2

After infestation of the five NFT calves with *R. microplus* larvae, the infection rate of *A. marginale* was 100% (5 out of 5). The presence of this rickettsia was confirmed by blood smears on different days after infestation, for each calf: days 15, 17, 16, 14, and 14, respectively. Additionally, *A. marginale* DNA was detected in the blood of all five NFT calves on day 14 according to PCR. By day 25 or day 36, all five NFT calves showed positive antibody titers against *A. marginale* by ELISA (Table 3). Between day 17 and day 25, a total of 3,358 engorged females were collected from the pen housing these five NFT calves. On day 25, salvage treatment against *R. microplus* was administered to the five calves due to an average tick burden of 183 ticks per calf. Additionally, each of these five calves required salvage treatment against *A. marginale* with enrofloxacin between day 17 and day 36. The PCV values for these calves ranged from 11% to 18%, while *A. marginale* bacteremia ranged from 3.4% to 16.4% (Table 3; Fig. 4). The two calves kept as negative controls remained uninfected by *A. marginale*, and they were not infested by ticks throughout the experimental period.

**Table 3:** Molecular detection of the surface protein 5 (*msp5*) gene of *Anaplasma marginale* and antibody titers (ELISA) in blood or serum samples collected from calves in Experiment 2, infested with the first and second generation of *Rhipicephalus microplus*

Calves used in the first step of the Experiment 2					
Group	Number of positive calves for *Anaplasma marginale* by PCR				
	Day − 119 up day 7	Day 14	Day 21	Day 28	Day 35
NFT	0/5	5/5	4/4	2/2	1/1
Negative control	0/2	0/2	0/2	0/2	0/2
Calf	Antibody titers against *A. marginale* by ELISA, for each calf				
	Day − 7 and day − 1		Day 17	Between Day 21 to 31	Day 36
35	Negative			1.176 *	
36	Negative				1.147 *
37	Negative			0.957 *	
40	Negative		1.254 *		
41	Negative			0.921 *	
NC1	Negative				Negative
NC2	Negative				Negative
Calves used in the second step of the Experiment 2					
Group	N of positive calves for *Anaplasma marginale* by PCR				
	Day − 119 up day 7	Day 14	Day 21	Day 28	Day 35
NFT	0/5	5/5	1/1	Not performed - Calves received salvage treatment before	
Negative control	0/2	0/2	0/2		
Calf	Antibody titers against *A. marginale* by ELISA, for each calf				
	Day − 7 and Day − 1		Between day 20 up to 24	Day 28	Day 35
146	Negative		1.032 *	Not performed - Calves received salvage treatment before	
148	Negative		0.374 *		
149	Negative		0.687 *		
142	Negative		1.264 *		
141	Negative		0.987 *		
NC1	Negative		Negative		
NC2	Negative		Negative		

* Calves received salvage treatment against *A. marginale*

NFT = Negative calf for *A. marginale* and tick-free until day 0 of the study

PWT = Positive calf for *A. marginale* and infested with *R. microplus* on day 0 of the study

NC = Negative control

#### Second tick generation

To proceed with the second step of Experiment 2, 224 engorged females from the first generation, which detached from calves 36 and 37 on day 24 of the study, were employed. On that day, these two calves exhibited mean *A. marginale* bacteremia values of 3.8% and 7.2%, respectively. Just like in step 1, the infection rate of *A. marginale* for the five new calves (examined through blood smears, PCR, and ELISA) by *R. microplus* larvae was 100% (5 out of 5). The infections were confirmed through blood smears on different days after infestation for each calf: 16, 17, 14, 18, and 15, respectively. Through PCR, all five NFT calves showed the presence of *A. marginale* DNA on day 14, and they all exhibited antibody titers against *A. marginale* at the end of the study (Table 3).

**Fig. 5:**
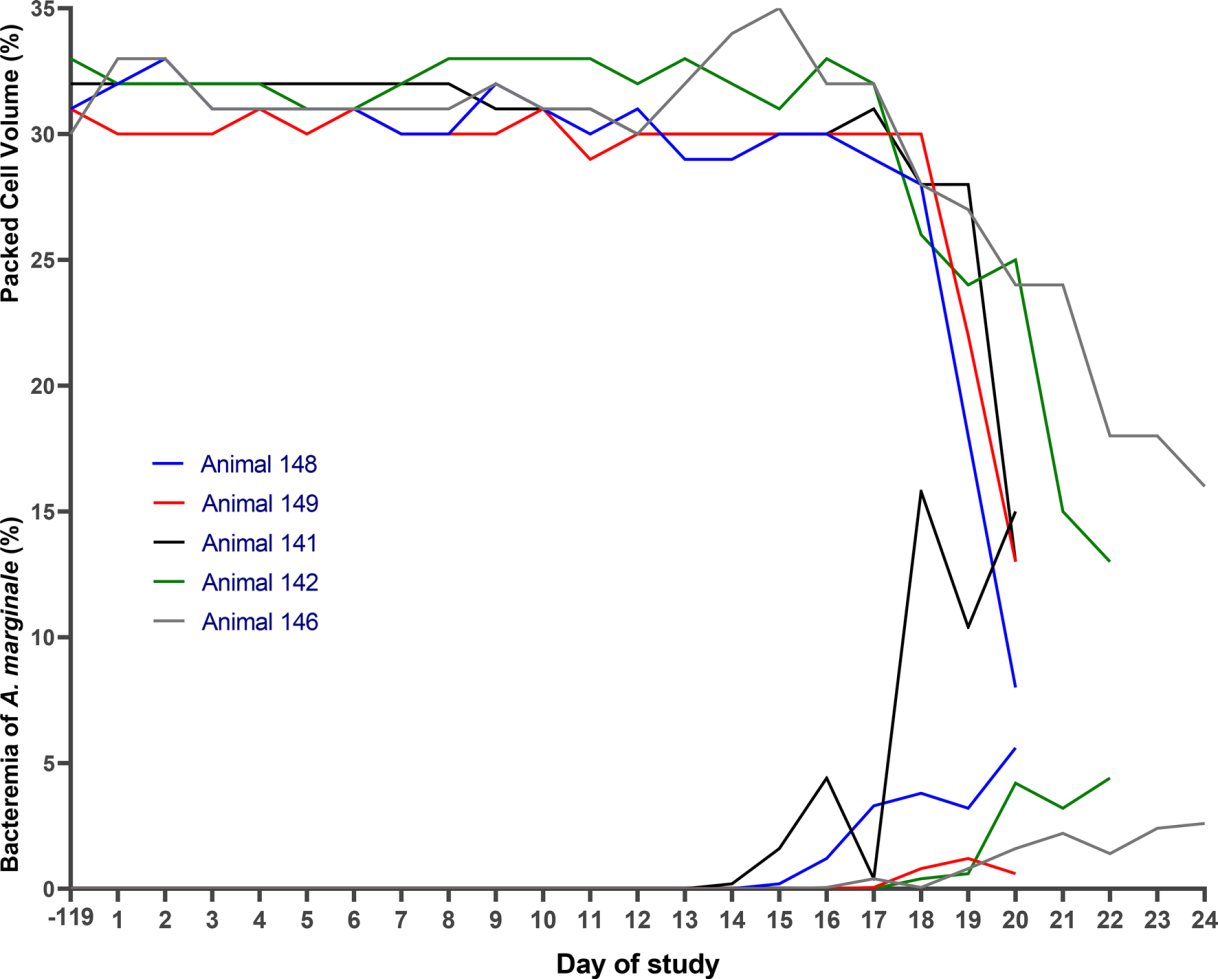
Experiment 2 - step 2: packed cell volume and *Anaplasma marginale* bacteremia in calves negative for *Anaplasma marginale*, infested with 40,000 larvae of the second generation of *Rhipicephalus microplus.* The engorged females were fed on calves that became naturally infected with *A. marginale* in step 1

On day 20, the five NFT calves received treatment to control cattle ticks due to a high load of *R. microplus* on the calves, with mean tick counts reaching up to 413.4 ticks per calf. Additionally, all five calves required salvage treatment against *A. marginale* on days 24, 20, 20, 22, and 20, respectively. On these days, the calves displayed PCV values ranging from 8% to 16%, along with bacteremia levels between 0.6% and 15% (Fig. 5).

In the fly traps positioned near the barn during both the first and second generations of the tick experiment, calliphorids, one specimen of *Haematobia irritans*, and phytophagous hemipterans were found.

## Discussion

This study offers valuable insights from an applied perspective regarding the infection rate of *A. marginale* by *R. microplus*. The findings confirm the infection of calves with *A. marginale* via intra-transstadial, and through unfed larvae of *R. microplus* originating from engorged females that had fed on animals naturally infected with *A. marginale*. These results contributed to a better of the epidemiological role that this tick plays in bovine anaplasmosis, especially in tropical regions where both anaplasmosis and this tick are prevalent.

### Transstadial and intrastadial transmission

Previous studies have documented transstadial and intrastadial transmission of *A. marginale* by *R. microplus* (Uilenberg 1970; Aguirre et al. 1994). In the present study, 70% (7 out of 10) of initially *A. marginale*-negative calves became infected during the experimental period. Although the *A. marginale* strain transmitted by ticks to calves was not molecularly characterized in the current Experiment 1, it was presumed to be the same strain infecting both. Nevertheless, the observed infection pattern is consistent with tick-mediated transmission, given that all other potential routes of infection were controlled and that negative control animals remained uninfected throughout the experimental period.

This high proportion of infected calves (70%) occurred despite a low rate of tick transfer between cattle (0.1%) and the fact that PWT cattle exhibited *A. marginale* bacteremia levels varying from 0.2% to 2.6%. In two NFT calves, only one *R. microplus* male was observed during 31 days. Even though this tick fed on calves with *A. marginale* bacteremia levels ranging from 0.4% to 1.4%, both NFT calves became infected. Conversely, two NFT calves had ticks but remained uninfected by *A. marginale*. Aguirre et al. (1994) observed that bacteremia levels exceeding 0.3%, combined with the presence of up to three ticks, were sufficient for *R. microplus* to transmit *A. marginale* to cattle via transstadial transmission. Those researchers found that *A. marginale* remained viable for 72 h in *R. microplus* when they were off the host during molting from nymphs to adults. Despite these observations, bacteremia levels are currently not considered a crucial factor for tick-mediated transmission of anaplasmosis to cattle. In contrast, the characteristics of specific *A. marginale* strains appear to be more important in this epidemiological context, as some strains are more readily transmissible by ticks than others (Scoles et al. 2007; De la Fournière et al. 2023).

The pre-patent period (PPP) observed in their study ranged from 22 to 35 days, which is consistent with the PPP of 14 to 32 days observed in the current study. The PPP for *A. marginale* in cattle can vary, depending on factors like host susceptibility, from 7 to 60 days (Kocan et al. (2010); Gale et al. 1996; Vidotto and Marana. 2001; Kocan et al. 2003).

The migration of *R. microplus* between cattle is a phenomenon previously reported in the literature (Guglielmone 1995; Mason and Norval 1981; Uilenberg 1970; Panizza et al. 2022a). A key observation in the present study was that like observed by Uilenberg (1970); Panizza et al. (2022a), both females and males can migrate between cattle. In a study by Masson and Norval (1981), calves were infested with 30,000 larvae, and after 24 h they were placed together with other calves without ticks. During the evaluation period, larvae and adult ticks were observed to move between the calves. In the present study, only adults (males and females) of *R. microplus* were observed migrating.

The rate of *R. microplus* migration among cattle can vary, ranging from 0.01 (Guglielmone 1995) to 0.1%, as observed in the present study over periods of eight and 31 days, respectively. At the herd level, as reported by Panizza et al. (2022a), 25% of cattle kept at 2 AU/hectare and 65% of cattle at 5 AU/hectare were infested. In the present study, 90% of the calves became infested. However, it is important to note that the stocking rate in the present study during the 31 days was 50.3 AU/hectare, higher than in typical field conditions. Both Panizza et al. (2022a) and we evaluated calves for relatively short periods, 13 and 31 days, respectively. In actual field conditions, calves often remain together for longer periods, typically months, increasing opportunities for encounters and physical contact between cattle. Consequently, this extended contact can elevate the rate of *R. microplus* passage between calves.

In practical terms, this indicates that even when an anaplasmosis outbreak is associated with intrastadial transmission by ticks, infected cattle may not present visible tick infestation, due to the extremely low rate of tick migration between animals. This characteristic is highly relevant under field conditions and should be considered when investigating outbreaks of anaplasmosis in which the cattle tick is involved (Leal et al. 2024). However, the low number of ticks migrating among cattle are sufficient to infect other calves with *A.* *marginale*. This underscores the epidemiological importance of these pathways for *A. marginale* in cattle, particularly in regions where *R. microplus* occurs.

### Transmission by unfed *R. microplus* larvae

In a study conducted in Colombia with a method like that described here, Lopez and Vizcaino (1992) infested calves with 20,000 larvae of *R. microplus*, originating from females of these ticks that had fed on cattle with *A. marginale* bacteremia of 7%. In that study, calves infested with larvae of the first and second generations were infected by *A. marginale*, with a pre-patent period (PPP) as early as 14 to 16 days after infestation. Similarly, De la Fournière et al. (2023) reported the presence of *A. marginale* in cattle after 22 days of infesting calves with 16,000 *R. microplus* larvae. These researchers also found the DNA of this rickettsia in the egg masses and larvae from females of *R. microplus*. The PPP results of these studies (Lopez and Vizcaino, 1992; De la Fournière et al. 2023) are very similar to the findings of the present study, where the PPP ranged from 14 to 18 days following infestation with 40,000 larvae per calf.

In the case of the study carried out by Panizza et al. (2022b), researchers used two splenectomized calves, which were infested with 10,000 larvae obtained from *R. microplus* females that had fed on cattle infected with *A. marginale* (bacteremia ≤ 1.3%). In this case, neither of the two calves evaluated by those authors became infected with the rickettsia in question. Similar results were observed with a calf in the studies by Ribeiro et al. (1996) and Ruiz et al. (2005). In a study by Ribeiro et al. (1996), engorged females fed on a calf with 4.3% *A. marginale* bacteremia. These females were maintained under field climatic conditions, although the number of larvae used to infest the susceptible cattle was not described by these researchers. In turn, Ruiz et al. (2005) reported that engorged females were fed on calves with 75% *A. marginale* bacteremia, using 6,000 larvae to infest susceptible splenectomized calves, but transmission did not occur.

Most of the previously cited experiments used between 10,000 and 20,000 *R. microplus* larvae per animal under stall conditions. Although the use of 40,000 larvae per animal in the present study may appear high compared with earlier reports, this number of larvae used in the presente study simulate field conditions in tropical regions. Andreotti et al. (2018) estimated the larval challenge in grazing Nelore and Brangus cattle. Animals were maintained in adjacent paddocks at a stocking rate of approximately 0.6 animal units per hectare (AU/ha). Using this model, larval exposure was estimated at approximately 27,108 larvae/day/animal for Nelore cattle and at least 76,119 larvae/day/animal for Brangus cattle. Based on these findings, the number of *R. microplus* larvae used per animal in the present study is consistent with field conditions. These values indicate that, under natural grazing conditions, cattle may be exposed to approximately 40,000 *R. microplus* larvae within about 1.5 days for Nelore cattle and less than one day for Brangus cattle, highlighting the high parasitic pressure to which hosts are subjected in tropical areas where this study was conducted.

The transovarian transmission of *A. marginale* in *R. microplus* has also been a subject of research, yielding varying results. Several studies, including those by Thompson and Roa (1978); Potgieter (1979); Ruiz et al. (2005); Esteves et al. (2015); Panizza et al. (2022b), found no evidence of this phenomenon. In contrast, Rosenbusch and Gonzalez (1923), Lopez and Vizcaino (1992), Estrada et al. (2020), and De la Fournière et al. (2023) all reported transovarian transmission. Moura et al. (2003) utilized molecular techniques, specifically nested PCR for the *msp5* gene, and detected *A. marginale* DNA fragments in egg masses and larvae of *R. microplus* that had fed on cattle with bacteremia ranging from 0.0% to 0.2%. Similarly, Shimada et al. (2004) employed PCR with the *msp5* gene and diagnosed *A. marginale* in larvae of *R. microplus* derived from engorged females that had fed on cattle with bacteremia ranging from 0.01% to 1%. However, the competence of these *R. microplus* larvae containing *A. marginale* DNA was not tested in calves in the studies performed by Moura et al. (2003) and Shimada et al. (2004). Estrada et al. (2020), using PCR to detect genes *msp5* and *msp1α* in larvae used to infest two calves, found DNA of *A. marginale*. These calves were subsequently infected by this rickettsia eight weeks after tick infestation following splenectomy. De la Fournière et al. (2023) described transovarial transmission of *A. marginale* in *R. microplus* ticks under natural conditions and the vector competence of infected *R. microplus* larvae in transmitting *A. marginale* to susceptible bovines during an experimental infection study. These authors also observed that *A. marginale* strains underwent bottlenecks during the transmission cycle, from a naturally infected bovine to the tick vector and its offspring, and then to a susceptible bovine.

Although transovarian transmission of *A. marginale* by *R. microplus* is still a matter of debate, in the present study, all the calves infested with first- and second-generation larvae of *R. microplus* (100%, 10 out of 10) became infected with *A. marginale* and needed treatment against this agent. It is evident that infection of calves with *A. marginale* through *R. microplus* unfed larvae has indeed occurred. This finding underscores the significant role of this transmission route in the epidemiology of bovine anaplasmosis in regions where this occurs. Another important point that should be highlighted, as discussed in Experiment 1, is that studies conducted to date suggest that the level of bacteremia in cattle infected with *A. marginale* is not the primary determinant of tick-mediated transmission. Instead, transmissibility appears to be strongly influenced by the specific *A. marginale* strain involved. Some strains seem to be more readily transmissible, such as the strain evaluated in the present study and that used by De la Fournière et al. (2023), whereas others, such as the Florida strain, are reportedly not transmissible (Scoles et al. 2007).

Regardless of whether infection by *A. marginale* occurred via the intra-transstadial route and by unfed larvae of *R. microplus*, the results of the present study, together with those found by De Moraes et al. (2024) and Morel et al. (2024), reaffirm that although other transmission routes of anaplasmosis exist, this tick species represents the primary determinant of *A. marginale* occurrence in cattle in subtropical and tropical regions where it is present. Outbreaks of bovine anaplasmosis frequently occur in tropical climate regions, and the results found in the present study help to clarify some of these situations. Examples of this are outbreaks numbered 4, 5 and 6 described in the work published by Leal et al. (2024), in which the bovine tick was involved. Probably one or all of the pathways described in that work are involved in these outbreaks.

## Conclusions

This work confirms the infection of calves with *A. marginale* via intra or transstadial and by unfed larvae of *R. microplus* routes, originating from engorged females that had fed on animals naturally infected with *A. marginale*. In this case, 70% of the calves became infected either through the intrastadial or transstadial route. Moreover, when calves were infested with unfed larvae of *R. microplus*, obtained from engorged females that had fed in calves with anaplasmosis, 100% of them were infected by *A. marginale*. These results show that *R. microplus* plays a significant role in the epidemiological transmission of *A. marginale.*

## Data Availability

The datasets generated during and/or analyzed in the current study are available from the corresponding author upon reasonable request.
